# Correction: Neuroinflammatory inhibitors from *Gardneria nutans* Siebold & Zuccarini

**DOI:** 10.1039/d1ra90146j

**Published:** 2021-09-16

**Authors:** Ying-Ying Si, Wei-Wei Wang, Qing-Mei Feng, Zhen-Zhu Zhao, Gui-Min Xue, Yan-Jun Sun, Wei-Sheng Feng, Young Jun Im, Xian-Shi Wang

**Affiliations:** College of Pharmacy, Henan University of Chinese Medicine Zhengzhou 450046 China; The Engineering and Technology Center for Chinese Medicine Development of Henan Province Zhengzhou 450046 China; College of Pharmacy, Chonnam National University Gwangju 500-757 61186 South Korea imyoungjun@jnu.ac.kr; School of Pharmaceutical Sciences, Wenzhou Medical University Wenzhou 325035 China xianshifree@126.com

## Abstract

Correction for ‘Neuroinflammatory inhibitors from *Gardneria nutans* Siebold & Zuccarini’ by Ying-Ying Si *et al.*, *RSC Adv.*, 2021, **11**, 27085–27091. DOI: 10.1039/D1RA05204G.

The authors regret that the first and last names for Young Jun Im were reversed in the original article. The corrected author list is shown above. The Royal Society of Chemistry apologises for these errors and any consequent inconvenience to authors and readers.

## Supplementary Material

